# Hospital-at-Home management of *Wickerhamiella pararugosa* candidemia and treatment with rezafungin: A case report

**DOI:** 10.1016/j.mmcr.2026.100827

**Published:** 2026-10-05

**Authors:** Inigo Vázquez Gomara, Javier Bernardo Perez-Serrano, Ma Auxiliadora Semiglia Chong, Lidia Vilches-Miguel, Diego Real de Asua Cruzat, Pablo Rodriguez-Cortes

**Affiliations:** a Department of Internal Medicine, Hospital Universitario de La Princesa, Madrid, Spain; b Department of Microbiology, Hospital Universitario de La Princesa, Madrid, Spain; c Department of Cardiology, Hospital Universitario de La Princesa, Madrid, Spain; d PhD Programme in Medicine and Surgery, Universidad Autonoma de Madrid, Madrid, Spain

**Keywords:** *Wickerhamiella pararugosa*, Candidemia, Rezafungin, Hospital-at-Home, Takotsubo syndrome

## Abstract

*Wickerhamiella pararugosa*, a rare cause of bloodstream infection, was identified in an 87-year-old woman admitted to a Hospital-at-Home unit for a complicated urinary tract infection, without a central venous catheter. Empirical micafungin was switched to rezafungin, a long-acting echinocandin, enabling HaH-based completion of therapy. Work-up revealed Takotsubo syndrome, managed without disrupting antifungal treatment. This is the first reported HaH management of this species and the first use of rezafungin for *W. pararugosa* infection.

## 1 Introduction

*Wickerhamiella pararugosa* (formerly *Candida pararugosa*) is an ascomycetous yeast only rarely implicated in invasive human disease. Reclassified from the genus *Candida* in 2017 [1], it has caused bloodstream infection in only ten previously reported patients, most with an indwelling central venous catheter and/or underlying malignancy [[2], [3], [4], [5], [6], [7], [8], [9], [10]]. No species-specific CLSI/EUCAST susceptibility breakpoints exist for *Wickerhamiella spp.*, and reported isolates show variable fluconazole and echinocandin minimum inhibitory concentrations [11].

We report a case of *W. pararugosa* candidemia without a pre-existing central venous catheter, managed within a Hospital-at-Home (HaH) program. The fully susceptible isolate was treated with rezafungin, a long-acting echinocandin. To our knowledge, this represents the first reported use of rezafungin and the first HaH-based management of bloodstream infection due to this species.

## 2 Case presentation

An 87-year-old woman with insulin-dependent type 2 diabetes mellitus, hypertension, dyslipidemia, and a history of high-grade papillary urothelial bladder carcinoma treated with radical cystectomy and ileal conduit urinary diversion presented to the Emergency Department (day 0) with fever and rigors. Blood cultures and a urine culture were obtained, and empirical intravenous ceftriaxone was initiated; she was admitted to the Internal Medicine ward.

By day +2, she had improved clinically, with resolution of fever and improving inflammatory markers. Abdominal ultrasonography showed no relevant abnormalities. The urine culture grew mixed flora >100,000 CFU/mL, and ceftriaxone was continued for a presumed complicated urinary tract infection associated with the ileal conduit. On day +3, after confirming she met HaH inclusion criteria, she was transferred to the HaH unit. She was functionally independent with adequate social support.

One of the two blood culture bottles drawn on day 0 became positive for yeast after 59 hours of incubation. The patient remained clinically stable, with no history of central venous catheterization or previous fungal isolation. Initial identification by matrix-assisted laser desorption/ionization time-of-flight mass spectrometry (MALDI-TOF MS) directly from the positive culture was unsuccessful; after 24 hours of further incubation, growth remained insufficient for reliable identification. A BioFire FilmArray Blood Culture Identification 2 (BCID2) panel (bioMérieux, Marcy l'Étoile, France) was subsequently performed but also failed to identify the organism.

Repeat blood cultures were obtained and empirical intravenous micafungin 100 mg once daily was started (day +3); also transthoracic echocardiography was requested as part of the candidemia work-up. Abdominal ultrasonography had already shown no relevant abnormalities. Fundoscopy was not performed, in accordance with an institutional risk-based protocol, as the patient was able to report symptoms, remained ophthalmologically asymptomatic and had no risk factors for ocular involvement 12,13.

By day +5 (approximately 60 hours after the blood culture became positive), sufficient growth was available for identification by MALDI-TOF MS, which identified the isolate as *Wickerhamiella pararugosa*. Susceptibility testing by SensititreTM YeastOneTM showed MICs of 4 μg/mL for fluconazole and 0.06-0.25 μg/mL for caspofungin, micafungin and amphotericin B. No species-specific CLSI/EUCAST clinical breakpoints are available, so susceptibility interpretation was based on available epidemiological cutoff values. Both the prolonged time to positivity and the slow growth on subculture are consistent with previous reports in this genus [8,14].

On day +6, given the absence of established evidence for a specific treatment regimen for this organism and the favourable susceptibility profile, antifungal therapy was switched from micafungin (after three days of treatment) to rezafungin (400-mg loading dose, followed by a 200-mg dose seven days later) to facilitate HaH administration.

Transthoracic echocardiography performed during the candidemia work-up unexpectedly showed regional wall-motion abnormalities (left ventricular ejection fraction of 41% without valvular vegetations) suggestive of Takotsubo cardiomyopathy. The patient was temporarily transferred to Cardiology for further evaluation. Coronary angiography excluded significant epicardial coronary disease, and ventriculography showed findings consistent with recovering Takotsubo syndrome. Mild signs of volume overload were also identified and were managed with low-dose loop diuretics.

Once clinically and analytically stable, the patient was readmitted to the HaH program to complete antifungal treatment. On day +13 (seven days after the loading dose), the scheduled second dose of rezafungin was administered. As repeat blood cultures obtained in the interim were already negative, antifungal therapy was considered complete. The patient was discharged from HaH in good clinical condition on day +15, with follow-up arranged with Cardiology and Primary Care.

## 3 Discussion

We present a case of fungemia caused by *W. pararugosa* treated with rezafungin in a HaH setting. Including the present case, eleven episodes of *W. pararugosa* bloodstream infection have been reported worldwide [[2], [3], [4], [5], [6], [7], [8], [9], [10]], with reported ages ranging from 6 months to 87 years. A central venous catheter and/or underlying malignancy was present in most previous cases; our patient differs in both respects, having no pre-existing vascular access. *Wickerhamiella* species are predominantly environmental yeasts recovered from flowers and pollinating insects [1], and only a few, mainly *W. pararugosa*, have been isolated from human clinical specimens 14. *W. pararugosa* was originally isolated from human faeces and has been recovered from the oral cavity, suggesting mucosal colonization as a plausible reservoir 15. A complicated urinary tract infection related to the ileal conduit was initially considered the most likely source; however, no yeasts were recovered from urine and, given this mucosal reservoir, gastrointestinal or peristomal translocation through the ileal conduit is a plausible alternative portal of entry. This broadens the recognized host spectrum for this rare pathogen beyond the classic oncology/catheter-related setting.

Identification of this species is challenging. Phenotypic systems have historically misidentified it as *C. rugosa*: among 24 clinical isolates phenotypically identified as *C. rugosa*, 10 were shown to be *C. pararugosa* by rDNA sequencing 16. Colonies on chromogenic agar are smooth and pale purple and are not distinctive [8], and *W. pararugosa* is not a target of the BCID2 panel. MALDI-TOF MS with an adequate reference library, or rDNA sequencing, is therefore required for reliable identification 17.

Antifungal susceptibility in this species is heterogeneous: most previously reported isolates, as well as the largest available in vitro survey, have shown elevated fluconazole MICs and, less consistently, reduced echinocandin susceptibility. Although rezafungin susceptibility was not directly tested, available in vitro studies including uncommon *Candida* species have shown rezafungin activity broadly comparable to that of micafungin and anidulafungin 18, supporting a reasonable extrapolation from the observed echinocandin susceptibility profile. In the absence of species-specific breakpoints, isolate-specific susceptibility testing remains essential.

Rezafungin's prolonged half-life, enabling once-weekly, front-loaded dosing, was non-inferior to caspofungin for candidemia and invasive candidiasis in the phase 3 ReSTORE trial 19. To our knowledge, this is the first reported use of rezafungin for *W. pararugosa* infection. Its dosing profile is particularly well suited to ambulatory or HaH management, as it markedly reduces the frequency of intravenous administrations compared with daily echinocandins, a logistical advantage that may help extend outpatient antifungal therapy to selected patients with invasive fungal infection. No previous report has described HaH management of candidemia due to this species; general outpatient parenteral antimicrobial therapy (OPAT) experience with antifungals remains more limited than with antibacterials, but supports feasibility in clinically stable, microbiologically characterized patients with adequate vascular access 20.

Takotsubo syndrome is a recognized, usually reversible complication of sepsis of any origin 21. We are not aware of a previous description specifically associated with candidemia; we report this temporal association as a secondary clinical observation rather than evidence of a fungus-specific mechanism, since obstructive coronary disease was excluded and the pattern and clinical course were consistent with sepsis-associated stress cardiomyopathy.(see Table 1)

**Table 1:** Reported cases of bloodstream infection due to *Wickerhamiella* (*Candida*) pararugosa (n = 11).

Reference/Year	Age/Sex	Main risk factor/source	Antifungal treatment	Outcome
Taj-Aldeen et al., 2014 [2]	6 mo/M	Intrauterine growth restriction; catheter not reported	Amphotericin B	Died*
Taj-Aldeen et al., 2014 [2]	5 y/M	Not reported; catheter not reported	Amphotericin B	Survived
Oliveira et al., 2014 [3]	Child (age/sex not reported)	Not reported	Not reported	Not reported
El Helou & Palavecino, 2017 [4]	39 y/F	PICC; parenteral nutrition; post-surgical necrotizing fasciitis	Micafungin + catheter removal	Survived
Noni et al., 2020 [5]	3 y/F	Acute lymphoblastic leukemia; catheter not reported	Not reported	Not reported
Piatti et al., 2021 [6]	61 y/F	Port-a-cath; breast cancer	Fluconazole + catheter removal	Died*
Nasri et al., 2023 [7]	3 y/M	Port catheter; acute myeloid leukemia	Caspofungin + catheter removal	Survived
Murata et al., 2024 [8]	80s/F	CVC; colorectal cancer with intestinal obstruction	Fosfluconazole - > micafungin	Survived
Najafzadeh et al., 2024 [9]	79 y/F	COVID-19; congestive heart failure; diabetes mellitus; catheter present (type unspecified)	Not reported	Died
Kang et al., 2025 [10]	62 y/F	Metastatic small cell carcinoma of endometrium; multiple embolic brain infarctions/possible IE; chemoport	Micafungin + chemoport removal	Died*
Present case, 2026	87 y/F	Presumed complicated UTI or peristomal/gastrointestinal translocation (Bricker ileal conduit); no pre-existing central venous catheter	Micafungin - > rezafungin	Survived

Abbreviations: UTI, urinary tract infection; CVC, central venous catheter; PICC, peripherally inserted central catheter; IE, infective endocarditis; MIC, minimum inhibitory concentration; ECV, epidemiological cutoff value; mo, months; y, years; M, male; F, female. * Died due to comorbidity progression.

This report has the inherent limitations of a single case. Conclusions regarding the safety and efficacy of rezafungin and HaH management for this species cannot be generalized beyond a carefully selected, clinically stable patient with a known-susceptible isolate. The contribution of fungemia to this patient's presentation is uncertain: fever resolved with ceftriaxone alone, and repeat blood cultures drawn on day +3, before the first antifungal dose, were negative, suggesting transient fungemia. Nevertheless, a single positive blood culture for yeast is never regarded as a contaminant, and treatment is recommended in all cases given the risk of clinically silent metastatic foci.

In conclusion, *W. pararugosa* candidemia can occur outside the traditional catheter or malignancy-associated setting and may show favourable antifungal susceptibility. In selected clinically stable patients, rezafungin's prolonged dosing interval facilitates HaH management of invasive infections caused by uncommon *Candida*-related species.

## CRediT authorship contribution statement

**Inigo Vázquez Gomara:** Writing – review & editing, Writing – original draft, Investigation, Data curation, Conceptualization. **Javier Bernardo Perez-Serrano:** Writing – review & editing, Writing – original draft, Investigation, Data curation, Conceptualization. **Ma Auxiliadora Semiglia Chong:** Writing – review & editing, Writing – original draft, Investigation, Data curation, Conceptualization. **Lidia Vilches-Miguel:** Writing – review & editing, Writing – original draft, Investigation, Data curation, Conceptualization. **Diego Real de Asua Cruzat:** Writing – review & editing, Writing – original draft, Investigation, Data curation, Conceptualization. **Pablo Rodriguez-Cortes:** Writing – review & editing, Writing – original draft, Visualization, Validation, Investigation, Data curation, Conceptualization.

## Ethical approval and patient consent

This case report was prepared in accordance with institutional guidelines for the publication of clinical case data. Written and signed informed consent for publication of this case report was obtained from the patient. Formal institutional ethics committee approval was not required for the publication of a single anonymized case report.

## Note on submission to medical mycology case reports

The statements above (funding, conflicts of interest, and patient consent) must be transcribed into the journal's own Ethical Form, downloadable from the submission site (www.editorialmanager.com/mmcr). The corresponding author should complete, sign, and upload the scanned (or digitally signed) form together with the manuscript; submissions are not considered complete without it.

## Declaration of generative AI and AI-assisted technologies in the manuscript preparation process

During the preparation of this work, the authors used Claude (Anthropic) to assist with formatting the manuscript according to the journal's template and adapting it to the journal's structural requirements. After using this tool, the authors reviewed and edited the content as needed and take full responsibility for the content of the published article.

## Funding

This research did not receive any specific grant from funding agencies in the public, commercial, or not-for-profit sectors.

## Conflicts of interest

The authors declare that they have no known competing financial interests or personal relationships that could have appeared to influence the work reported in this paper.

## References

[1] de Vega C., Albaladejo R.G., Guzman B. (2017). Flowers as a reservoir of yeast diversity: description of Wickerhamiella nectarea f.a. sp. Nov., and Wickerhamiella natalensis f.a. sp. nov. from South African flowers and pollinators, and transfer of related Candida species to the genus Wickerhamiella as new combinations. FEMS Yeast Res..

[2] Taj-Aldeen S.J., AbdulWahab A., Kolecka A. (2014). Uncommon opportunistic yeast bloodstream infections from Qatar. Med. Mycol..

[3] Oliveira V.K.P., Ruiz L. da S., Oliveira N.A.J. (2014). Fungemia caused by Candida species in a children's public hospital in the city of Sao Paulo, Brazil: study in the period 2007-2010. Rev. Inst. Med. Trop. Sao Paulo.

[4] El Helou G., Palavecino E. (2017). Candida pararugosa: first reported bloodstream infection in an adult. Cureus.

[5] Noni M., Stathi A., Velegraki A. (2020). Rare invasive yeast infections in Greek neonates and children, a retrospective 12-Year Study. J. Fungi.

[6] Piatti G., Feltrin S., Fellini E. (2021). Catheter-Related sepsis by Candida pararugosa in an adult patient under chemotherapy regimen. Case Rep Infect Dis.

[7] Nasri E., Vaezi A., Shelerangkon M. (2023). Catheter-associated blood stream infections due to Candida pararugosa in a patient with acute myeloid leukemia: a case report. Braz. J. Microbiol..

[8] Murata S., Mimura K., Kawamura T. (2024). Bloodstream infection caused by Wickerhamiella pararugosa in a patient with intestinal obstruction: a case report. J. Infect. Chemother..

[9] Najafzadeh M.J., Shaban T., Zarrinfar H. (2024). COVID-19 associated candidemia: from a shift in fungal epidemiology to a rise in azole drug resistance. Med. Mycol..

[10] Kang T., Yun S.G., Nam M.H. (2025). First case of catheter-related bloodstream infection caused by Wickerhamiella pararugosa in Korea. Lab Med Online.

[11] Perez-Hansen A., Lass-Florl C., Lackner M. (2019). Antifungal susceptibility profiles of rare ascomycetous yeasts. J. Antimicrob. Chemother..

[12] Pappas PG, Kauffman CA, Andes DR (2016). Clinical Practice Guideline for the Management of Candidiasis: 2016 Update by the Infectious Diseases Society of America. Clin Infect Dis.

[13] Breazzano MP, Bond JB, Bearelly S (2022). American Academy of Ophthalmology Recommendations on Screening for Endogenous Candida Endophthalmitis. Ophthalmology.

[14] Takei S, Teramoto K, Fujimura J (2024). Isolation and identification of Wickerhamiella tropicalis from blood culture by MALDI-MS. Front Cell Infect Microbiol.

[15] Giammanco GM, Melilli D, Pizzo G (2004). Candida pararugosa isolation from the oral cavity of an Italian denture wearer. Res Microbiol.

[16] Paredes K, Sutton DA, Cano J (2012). Molecular Identification and Antifungal Susceptibility Testing of Clinical Isolates of the Candida rugosa Species Complex and Proposal of the New Species Candida neorugosa. Journal of Clinical Microbiology.

[17] De Carolis E, Vella A, Vaccaro L (2014). Development and Validation of an In-House Database for Matrix-Assisted Laser Desorption Ionization–Time of Flight Mass Spectrometry-Based Yeast Identification Using a Fast Protein Extraction Procedure. J Clin Microbiol.

[18] Winkler ML, Rhomberg P, Klauer AL (2024). The In Vitro Activity of Rezafungin Against Uncommon Species of Candida. Mycoses.

[19] Thompson GR, Soriano A, Cornely OA (2023). Rezafungin versus caspofungin for treatment of candidaemia and invasive candidiasis (ReSTORE): a multicentre, double-blind, double-dummy, randomised phase 3 trial. Lancet.

[20] van de Peppel RJ, Schauwvlieghe A, Van Daele R (2020). Outpatient parenteral antifungal therapy (OPAT) for invasive fungal infections with intermittent dosing of liposomal amphotericin B. Med Mycol.

[21] Cappelletti S, Ciallella C, Aromatario M (2017). Takotsubo Cardiomyopathy and Sepsis. Angiology.

